# ERAIZDA: a model for holistic annotation of animal infectious and zoonotic diseases

**DOI:** 10.1093/database/bav110

**Published:** 2015-11-18

**Authors:** Teresia M. Buza, Sherman W. Jack, Halid Kirunda, Margaret L. Khaitsa, Mark L. Lawrence, Stephen Pruett, Daniel G. Peterson

**Affiliations:** ^1^Department of Basic Sciences, College of Veterinary Medicine, Mississippi State University, Mississippi State, MS 39762 USA,; ^2^Institute for Genomics, Biocomputing & Biotechnology (IGBB), Mississippi State University, Mississippi State, MS 39762 USA,; ^3^Department of Pathobiology and Population Medicine, College of Veterinary Medicine, Mississippi State University, Mississippi State, MS 39762 USA and; ^4^National Livestock Resources Research Institute (NaLIRRI), Tororo, Uganda

## Abstract

There is an urgent need for a unified resource that integrates trans-disciplinary annotations of emerging and reemerging animal infectious and zoonotic diseases. Such data integration will provide wonderful opportunity for epidemiologists, researchers and health policy makers to make data-driven decisions designed to improve animal health. Integrating emerging and reemerging animal infectious and zoonotic disease data from a large variety of sources into a unified open-access resource provides more plausible arguments to achieve better understanding of infectious and zoonotic diseases. We have developed a model for interlinking annotations of these diseases. These diseases are of particular interest because of the threats they pose to animal health, human health and global health security. We demonstrated the application of this model using brucellosis, an infectious and zoonotic disease. Preliminary annotations were deposited into VetBioBase database (http://vetbiobase.igbb.msstate.edu). This database is associated with user-friendly tools to facilitate searching, retrieving and downloading of disease-related information.

Database URL: http://vetbiobase.igbb.msstate.edu

## Introduction

The 21^st^ century continues to be the era of big data involving not only the omics technologies but also applies to epidemiology (1–3) and public health (4–9) fields. Traditionally, the large volumes of structured or unstructured data generated from different fields are independently deposited into discipline-specific resources for use by the intended communities. Knowledge gaps result across related fields due to lack of connectivity in managing distinct but interrelated data. Take, e.g., the impact caused due to lack of knowledge of the emerging and reemerging animal infectious and zoonotic diseases (ERAIZD). It is clearly understood that ERAIZD continue to pose major threats to animal health, human health and global health security (10–14). To narrow the knowledge gaps and accelerate wide-ranging discoveries from interrelated data, it is essential that data integration is done first. As the ERAIZD-related big data continue to accumulate, there is an urgent need for an open-source unified resource associated with user-friendly tools to facilitate data usage. The utility of trans-disciplinary integrated disease data allows investigators to quantify key characteristics such as incubation periods, heterogeneity in transmission rates, duration of infections and the existence of high-risk groups (2).

At the epidemiological level, there have been several efforts to create global enhanced systems that address ERAIZD including sharing of health risk information at the animal–human–ecosystem interface. Global early warning and response system (GLEWS+) integrates information from the Food and Agriculture Organization (FAO) of the United Nations, World Organization for Animal Health (OIE) and the World Health Organization (WHO) Global Alert and Response (15, 16). Another early warning system is the Program for Monitoring Emerging Diseases (ProMED)-mail, an internet-based reporting program of the International Society for Infectious Diseases (ISID), which is dedicated to rapid global dissemination of up-to-date expert curated information on outbreaks of infectious diseases (17–19). The ProMed community obtains information from multiple sources including media reports, official reports, online summaries, local observers, etc. These reports are screened, reviewed and validated by in-country infectious disease experts before posting to the website. Additionally, agencies such as the Centers for Disease Control and Prevention (CDC) (20), the leading federal public health agency in the United States and the associated global disease detection program (21) provide invaluable information for public use. Moreover, a joint initiative of FAO and OIE established the Global Framework for progressive control of Transboundary Animal Diseases (22) to empower regional alliances in the fight against international spread of animal diseases.

At the molecular level, various infectious agents have different genetic makeups that may cause similar or different signs and symptoms. The molecular diagnosis of infectious diseases using nucleic acid-based technologies such as the polymerase chain reaction, ligase chain reaction, transcription-mediated amplification and nucleic acid sequence-based amplification have become tools in understanding key molecular factors related to ERAIZD causal agents. These techniques provide highly accurate diagnosis of infections caused by bacteria, viruses, fungi, parasites and others (23–28). For example, the CDC has been using advanced molecular detection technology, such as whole-genome sequencing as means of surveying the genetic differences between isolates in various pathogenic organisms. This has led to the establishment of a public database for sharing information about potentially deadly diseases such as anthrax and brucellosis (2). This type of genomic surveillance is more accurate, reliable and a more cost effective means of diagnosing known, emerging and reemerging infections, as well as characterizing foreign or unknown subtypes. However, these molecular techniques only demonstrate the presence of pathogen (or subtypes) and not the presence of disease.

Emerging and reemerging diseases are caused by diverse range of biological agents that exit their reservoirs, enter susceptible hosts through different routes and cause tissue damage. Provision of a resource that links findings from epidemiological and basic research investigations could help answer fundamental questions regarding factors responsible for disease development and so forth. We have developed a model for annotating multidimensional diverse ERAIZD data from a large variety of sources and integrating it into an open-access unified resource we call ERAIZDA (Emerging and Reemerging Animal Infectious and Zoonotic Disease Annotations). The model is referred to as ‘ERAIZDA model’ throughout the article. We envision that availability and utility of an ERAIZDA resource will (i) promote global data sharing beyond usual boundaries which could lead to improved interactions in multi- and interdisciplinary research teams; (ii) furnish the animal/human health and veterinary research communities with integrated disease information for developing effective joint policies and guidelines for controlling animal infectious and zoonotic diseases; (iii) lead to better planning of coordinated research strategies including setting up research priorities, developing testable data-driven hypotheses and identification of effective and efficient integrative interventions and (iv) lead to establishing database and data sharing standards used by multi- and interdisciplinary communities for collaborative data sharing, solving much of the current problems in data curation and database application programming interface and web services among related data sources. We strategically selected brucellosis to demonstrate implementation of the ERAIZDA model. Globally, brucellosis is one of the most significant zoonotic diseases (29) with public health challenge and economic impact. It has a worldwide distribution and is absent in only few countries (30). It is listed in the OIE (31, 32) as one of the 2015 notifiable diseases and in the WHO as one of the seven neglected endemic zoonoses (33).

## Structure of ERAIZDA model

**Figure 1.**
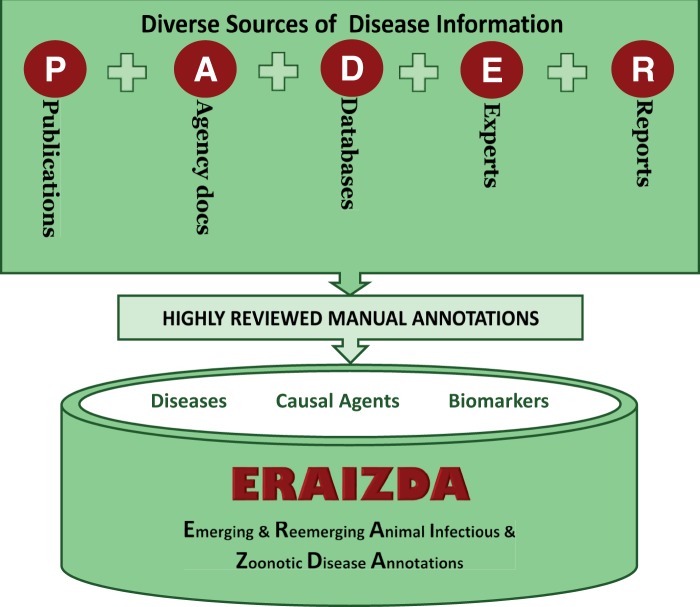
Sources of disease annotations. PADER is an abbreviation for P: Publications; A: Agency; D: Databases; E: Experts; R: Reports. ERAIZDA is an abbreviation for Emerging and Re-emerging Animal Infectious and Zoonotic Disease Annotations.

The ERAIZDA model adopts an annotation approach that takes into consideration all sources of information for an infectious disease that occurs at the animal–human–pathogen–ecosystem interface. Identification of information sources that collectively provide animal infectious, and zoonotic diseases is a first step when implementing this model. Five main sources of disease information were identified and designated as PADER (Figure 1), where P = publications (articles in journals, books or conference proceedings), A = agencies documents (from WHO, CDC, FAO, OIE, GLEWS+, ISID, etc.), D = databases [such as National Center for Biotechnology Information (NCBI), WAHID, Ontologies and host–pathogen interaction], E = expert validated information (from health departments, veterinary professionals, reviewed ProMed-mails, reviewed health news, educational resources, etc.) and R = reports officially documenting disease information (non-electronic reports). Each individual source is then manually annotated by highly skilled biocurators to generate associations describing the disease features, causal agents and molecular data such as biomarkers. These annotations are then organized and deposited into a unified resource.

**Figure 2.**
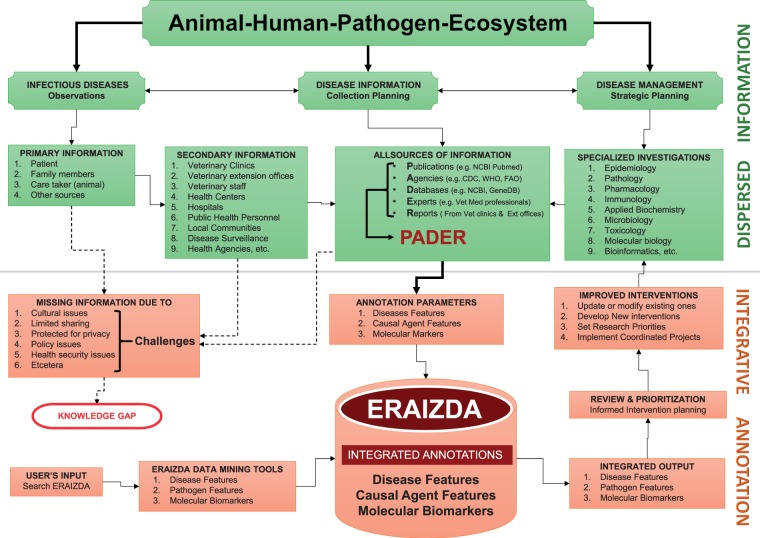
Structure depicting complete ERAIZDA model. The upper panel (green) shows part of the model depicting major key players in the disease management, planning process and sources of information. The lower panel (red) shows the disease annotation process and user interface.

Comprehensive annotation of animal infectious and zoonotic diseases using the PADER approach is a complex process that involves coordinated and dedicated effort of diverse expertise and skills to integrate multidisciplinary disease information into a unified resource. The complete ERAIZDA model herein described (Figure 2) demonstrates such involvedness. Briefly, we assumed that once a disease is observed in a community, the process of collecting the disease information and reporting of the incidence starts immediately. This is followed by disease management strategic planning by all key players including health personnel, epidemiologists and clinical or basic scientists who may conduct individual, multidisciplinary or interdisciplinary investigations on the disease. Traditionally, findings from these investigations are documented in different PADER sources. Annotation of these diverse sources could yield varied disease information that can be integrated into a unified ERAIZDA resource. Provision of user-friendly tools facilitates searching, retrieving and/or downloading specific information from the ERAIZDA resource. This information can then be thoroughly reviewed and prioritized to facilitate better informed decision making for new interventions or improving the existing ones.

## Annotation parameters

The ERAIZDA model is designed for comprehensive annotation of animal infectious and zoonotic diseases from diverse sources (PADER) to generate multidisciplinary disease-related information. We have defined parameters to be used as reference to guide the annotation process in each PADER source. These parameters are grouped into three broad categories representing (i) diseases, (ii) pathogens (causal agents) and (iii) molecular information, specifically disease biomarkers. The model adopts different terminologies for describing infection, frequency and typology of an infectious or zoonotic disease (Table 1). A detailed description of all parameters is given (see Supplementary File S1). We acknowledge that debate may exist around the description of these parameters which could lead to better and standardized disease annotation terms.

**Table 1.** Terminologies adopted by ERAIZDA model for classifying infectious and zoonotic diseases

Terminology	Description
**Infection type**	
Infectious (ID)	A disease caused by transmissible agents that can be spread directly or indirectly from one animal to another.
Zoonotic (IZD)	A disease caused by transmissible agents that can naturally be transmitted from animals to humans or humans to animals.
Zoonotic potential (IZP)	A disease caused by transmissible agents with a potential of becoming zoonotic but the importance of zoonotic transmission not fully known.
**Disease frequency**	
Endemic	A disease that occurs in a population with predictable regularity. The events are clustered in space but not in time.
Sporadic	A disease that occurs at irregular intervals in a few places; scattered or isolated
Epidemic/Epizootic	A disease that occurs in a population in excess of its normally expected frequency of occurrence. The events are clustered in time and space.
Pandemic	An epidemic of infectious disease that has spread through large populations across a large area covering ether multiple continents or the world
**Disease Typology**	
Type I	Diseases that occur in both developed and developing countries, with large numbers of vulnerable populations in each
Type II	Diseases that occur in both developed and developing countries, but with a substantial proportion of the cases in developing countries
Type III	Diseases that overwhelmingly or exclusively occur in developing countries

## Implementation of the ERAIZDA model

### Defining disease parameters

Brucellosis was chosen as a classical example of a significant endemic zoonotic disease to demonstrate the implementation of the ERAIZDA model. The annotations of brucellosis described herein are not by any means exhaustive but serve as model for annotating animal infectious and zoonotic diseases from diverse sources.

Preliminary annotation of selected PADER sources enabled us to establish important parameters (Table 2, Supplementary File S1) for guiding comprehensive annotation of animal infectious and zoonotic diseases using this model. In order to provide structured annotations the parameters were organized in an Excel spreadsheet where rows represented diseases and columns represented parameters.

**Table 2.** Parameters for guiding annotation of animal infectious and zoonotic diseases

**A: Disease parameters**	17. Treatment	33. Reservoir
1. Disease name	18. Preventive measures	34. Entry and exit portal
2. Name synonyms	**B: Pathogen parameters**	35. Hosts
3. Ontology (DO) name	19. Family	36. Transmission
4. DO identifier	20. Genus	37. Incubation (days, months)
5. Listing agency	21. Species	38. First isolation (year)
6. Causal agent	22. Species taxons (NCBI counts)	**C: Molecular parameters**
7. Type of infection	23. Subspecies	39. Biomarker name
8. Animal symptoms	24. Subspecies taxons (NCBI counts)	40. Biomarker symbol
9. Human symptoms	25. TaxonID	41. Biomarker UniProtKB AC
10. Outbreaks	26. Taxon level	42. Biomarker group
11. Distribution	27. Infectivity	43. Biomarker class
12. CMH disease type	28. Pathogenicity	44. Experimental organism
13. Disease frequency	29. Virulence	45. Biomarker references
14. Case fatality rate	30. Toxigenicity	46. Reference publication date
15. Risk factors	31. Resistance	47. Biomarker evidence text
16. Diagnosis	32. Antigenicity	48. URL for molecular data

CMH, Commission on Macroeconomics and Health.

### Google first-pass analysis

**Figure 3.**
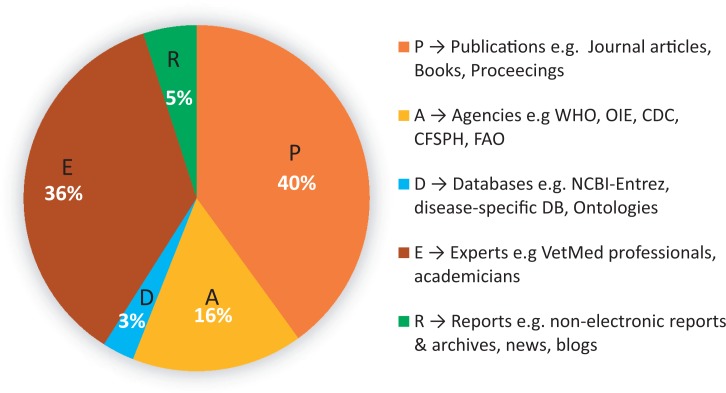
Clusters of top 10 google first-pass hits of brucellosis. Search terms included [brucellosis OR (brucellosis) OR (brucellosis zoonotic disease) OR (brucellosis in animal) OR (brucellosis in human)].

The process of annotation started by searching important keywords [brucellosis OR (brucellosis) OR (brucellosis zoonotic disease) OR (brucellosis in animal) OR (brucellosis in human)] in Google as First-Pass Analysis to get a clear picture about the expected sources of information and initial knowledge of brucellosis. The search returned 180 000 google hits. Clustering of the top 100 hits using PADER codes identified the most important sources of brucellosis information (Figure 3; Supplementary File S2).

From the First-Pass Analysis, we were able to generate preliminary brucellosis information that enabled us establish custom terms (values) for describing disease parameters (Supplementary File S1). In the process of annotating brucellosis we found that some parameters may not apply or be relevant to some diseases and data may not be available for some parameters. In such cases, the corresponding fields are represented with triple hyphen (—) until such data become available in the subsequent updates.

### Quantifiable sources of brucellosis information

The primary quantifiable sources of reliable brucellosis information, including epidemiology of the disease and molecular characterization of the causal agents, are a combination of articles published in peer-reviewed journals and controlled databases represented in the NCBI. The NCBI PubMed database is the most famous gateway for browsing information published in scientific journals. For example, searching the PubMed database (as of 5 May 2015) using brucellosis-related keywords [(brucellosis OR (brucellosis) OR (brucellosis zoonotic disease) OR (brucellosis in animal) OR (brucellosis in human) OR (Malta fever) OR (Mediterranean fever) OR (undulant fever)] retrieved 16 845 articles, including nine articles (34–42) that were published in the 19th century (Supplementary File S3). In the past 15 years, the number of brucellosis articles has been increasing progressively. A dedicated effort to annotate these articles can yield a very significant information that may provide insight of new ways for managing the brucellosis.

### Causal agents of brucellosis

Preliminary annotation of few selected articles indicated that *Brucella melitensis* was the first brucellosis causal agent to be characterized. Sir David Bruce isolated *Micrococcus melitensis* (now *B.* *melitensis*) from spleen of a British soldier who died from Malta fever (brucellosis) in Malta in 1886 (43). Subsequently, other *Brucella* species that infect animals have since been identified, including *Brucella abortus, Brucella suis, Brucella ovis, Brucella canis, Brucella pinnipedialis, Brucella ceti, Brucella neotomae, Brucella microti* and *Brucella inopinata* (44) (Supplementary File S4).

Currently, over 500 subspecies have been classified under these species and documented in the NCBI Taxonomy Database (44). *B.* *abortus* is the most characterized species as evidenced by the number of subspecies represented in the NCBI Taxonomy Databases and has more open-access PubMed articles (Supplementary File S5).

## Molecular data of brucellosis causal agents

### Entrez records

Entrez is the NCBI's primary text search and retrieval system that integrates diverse databases such as PubMed and Taxonomy databases with molecular databases such as DNA and protein sequences, genes, genomes, single-nucleotide polymorphs and gene expression data (45). Traditionally, each species represented in the NCBI Taxonomy Database is identified with a unique name and a taxon-specific unique identifiers that distinguish one species from the other. Unique names and identifiers of the *Brucella* species facilitated identification of species-specific molecular records of the causal agents of brucellosis (Supplementary File S6). These molecular data, especially the nucleotide (transcripts) and amino acid (protein) sequences, are very important in identifying unique features of indistinguishable causal agents. The ERAIZDA model includes a link for accessing most current molecular data for each annotated causal agents indexed in the Entrez databases.

### Preliminary biomarkers of brucellosis

The preliminary data of brucellosis biomarkers were annotated from a sample of only 10 PubMed central articles (Table 3) for demonstration. The complete biomarker annotations including experimental texts that support the annotation is available as Supplementary File S7. This data can also be retrieved from the VetBioBase database (46) using the dseMARKERS tool.

**Table 3.** Example of experimental-based biomarkers of brucellosis

Biomarker name	Symbol	Importance	*Brucella* species	PubMed ID
Zinc-dependent metallopeptidase	BAB1_0270	Virulence factor	*B. abortus*	PMID:24928771
Hypothetical protein	BAB1_0267	Virulence factor	*B. abortus*	PMID:24928771
Nucleoside diphosphate kinase	NDP	Vaccine candidate	*B. abortus*	PMID:25724777
Phosphoribosylamine-glycine ligase	purD	Mutant	*B. abortus*	PMID:25546140
Amidophosphoribosyltransferase	purF	Mutant	*B. abortus*	PMID:25546140
ATP-binding/permease protein	cydC	Mutant	*B. abortus*	PMID:25253663
ATP/GDP-binding protein	looP	Mutant	*B. abortus*	PMID:25253663
Ribosomal protein L9	L9	Vaccine candidate	*B. abortus*	PMID:23913725
mir-1981	mir-1981	Gene regulator	*B. melitensis*	PMID:22904669
Histidinol dehydrogenase	hisD	Drug target	*B. suis*	PMID:17481905
Histidinol dehydrogenase	hisD	Drug target	*B. suis*	PMID:17698620
Beta-carbonic anhydrase	bsCA I	Drug target	*B. suis*	PMID:20211561
Beta-carbonic anhydrase	bsCA II	Drug target	*B. suis*	PMID:21251841

## Integration of brucellosis information into unified resource

**Figure 4.**
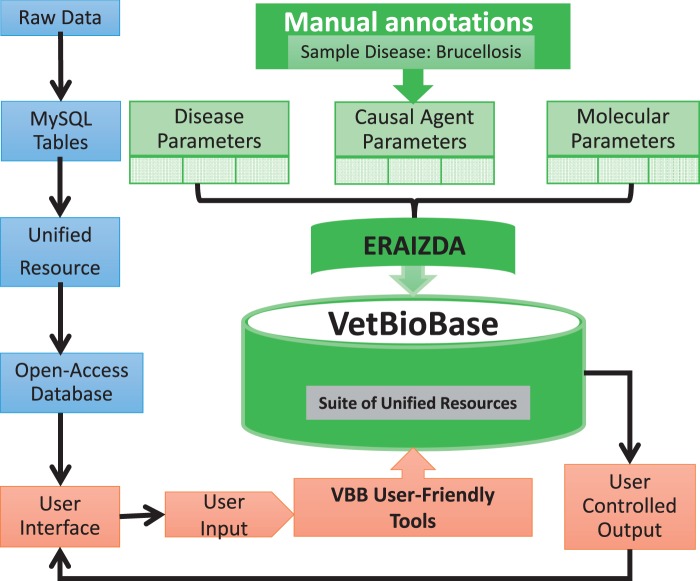
Structure for integrating ERAIDA into VetBioBase.

To facilitate management and sharing of preliminary annotations of brucellosis, we organized the raw data into MySQL relational tables representing disease features, causal agents and molecular biomarkers (Figure 4). Collectively, these tables formed a foundation for establishing ERAIZDA, a unified resource for integrating animal infectious and zoonotic disease annotations. The tables were then loaded into the VetBioBase database (46) for public use. These preliminary annotations enabled us to develop seven user-friendly tools for searching, retrieving and/or downloading specific information from the ERAIZDA dataset.

## Discussion

We present the ERAIZDA model, a comprehensive model for annotating animal infectious and zoonotic diseases. This model uses over 50 pre-defined parameters (Supplementary File S1) as a reference to guide the disease annotation process from diverse sources referred (in this article) as PADER. Each parameter describes significant information related to a particular infectious and/or zoonotic disease. The model encourages data integration into an open-access unified resource for use by the animal/human and veterinary research communities to accelerate integrative translational interventions aimed at achieving better understanding of infectious and zoonotic diseases. Using brucellosis as an example, we demonstrated that multidisciplinary disease-related information from epidemiological investigations, clinical studies, case reports and basic science functional genomics investigations can be linked together and made available through a single resource. Preliminary annotations of brucellosis were used as a foundation to create a unified resource known as ERAIZDA. Our future focus is to use the ERAIZDA model to generate annotations of all animal infectious and zoonotic diseases of national and international priority, starting with the notable diseases listed by the World Organization for Animal Health (OIE).

The central proposition of integrating animal disease data is to enable all key players in the disease management process to quickly access disease information and process it to uncover hidden value, narrow the knowledge gap and make data-driven decisions. Using the pre-defined parameters (Supplementary File S1) as reference is the most feasible way to ensure that every piece of available information is represented. It is most likely that a single source of disease information would not be able to provide all the parameters (diseases, causal agents, molecular biomarkers, etc.). This is why PADER becomes such an important approach. For example, when annotating molecular biomarkers using the ERAIZDA model, we recommend including supporting evidence. The best evidence is to link the biomarker with a reference such as PubMed articles and text that will enable users to have confidence in the annotated biomarker. For instance, sequences with amino acid replacement may signify useful molecular biomarkers for detecting mutations that could also be basis for detecting antimicrobial resistance and virulence determinants. Adding reference to the sequence information is particularly important, especially if the annotation involves cross referencing between related diseases. Here is an example of cross-species annotation from two articles; PubMed ID 21151656 (PubMed Central ID PMC2997342) and PubMed ID 17021120 (PubMed Central ID PMC1594805): ‘*Mycobacterium bovis*, a bacterium that causes bovine tuberculosis is naturally resistant to pyrazinamide (47) due to its inability to produce pyrazinamidase enzyme needed to convert pyrazinamide into active form of the antimicrobial agent (48). However, point mutations in the rpoB (DNA-directed RNA polymerase subunit beta) and katG (catalase-peroxidase) genes are considered potential biomarkers for resistance to rifampicin and isoniazid in human *Mycobacterium tuberculosis* (49)’. Adding a short supporting text like this enables researchers to quickly consider what to do next. In this example, researchers may use the resistance features to distinguish isolates of *M. bovis* from *M. tuberculosis*.

Although ERAIZDA model intends to link disease names with Disease Ontology terms, we are aware that there are other ontologies that are relevant to the ERAIZDA model. Since data generated using the ERAIZDA model is intended to benefit not only the research community but also a wide range of animal and human health-based communities, we have been very careful not to link ontological terminologies that are not familiar to the audience. Indeed, the model is expected to provide very specific quantitative data guided by pre-defined parameters (as described in Supplementary File S1) that can be used by ontology developers to improve the existing ontologies such as pathogen Transmission Ontology and Symptom Ontology or designing new ontologies. The model also integrates some aspects of host-pathogen interactome features related to the pathogen and hosts at molecular level. For example, we understand that virulence of a pathogen is one of possible effects of host-microbe interaction. In that case, a microbial virulence could depend on host factors to be effective. This is why the model links possible molecular biomarkers including virulence factors with pathogen characteristics to ascertain the effects of interaction on, e.g., the microbial virulence or pathogenicity in relation to effects induced by host genes.

It should be emphasized that the integrated data by itself is not useful, unless it is freely accessible by the intended communities for further interventions. User-friendly tools are provided to offer such access to the interested communities. Generally, the ERAIZDA model encourages multidisciplinary collaboration of all key players involved in the disease management process to share research and non-research data without their usual boundaries. It is anticipated that the ERAIZDA data will continue to grow progressively and sustainably as more animal infectious disease annotations are added, and the research community will utilize and benefit from this data. Although curation is one of the most likely challenging aspects of maintaining a sustainable ERAIZDA database, having a uniform annotation procedure can lessen the challenge. To maintain accuracy and uniformity of annotations, curators are encouraged to use special forms containing the pre-defined parameters (Supplementary File S1) to guide the annotation process in each PADER source. Having a standard annotation procedure will also facilitate data integration and updates.

## Conclusion

Annotation of animal infections and zoonotic diseases from diverse sources is a central initiative to reveal important information for new interventions. Most importantly, integrating the disease annotations into an open-access unified resource and provision of user-friendly tools for searching, retrieving and downloading specific information could save users considerable time and effort. We expect to increase the depth and breadth of the ERAIZDA dataset by continuing to annotate additional animal infectious diseases of national and international priority. We believe that since zoonoses can infect both animals and humans, both the medical, public health, basic research and veterinary communities will definitely benefit from this unified resource. We encourage interested communities to use the ERAIZDA data to develop additional models needed to understand the mechanism and pathology of animal infectious and zoonotic diseases and translation of functional genomics of infectious diseases into biological value.

## Supplementary data

Supplementary data are available at *Database* Online.

Supplementary Data
