# Supplementary material for: ERAIZDA: a model for holistic annotation of animal infectious and zoonotic diseases
Source: Database (Oxford). 2015 Nov 18;2015:bav110. doi: 10.1093/database/bav110 (PMC4651161; doi:10.1093/database/bav110)
Supplement: Supplementary Data [file supp_2015_bav110_index.html]

Supplementary Data 

# ERAIZDA: a model for holistic annotation of animal infectious and zoonotic diseases

## Supplementary Data

files

- Supplementary Data - zip file
