# Supplementary material for: ERAIZDA: a model for holistic annotation of animal infectious and zoonotic diseases
Source: Database (Oxford). 2015 Nov 18;2015:bav110. doi: 10.1093/database/bav110 (PMC4651161; doi:10.1093/database/bav110)
Supplement: Supplementary Data [file supp_bav110_suppl_data.zip › SupplemetaryFile_4.pptx]

## Slide 1
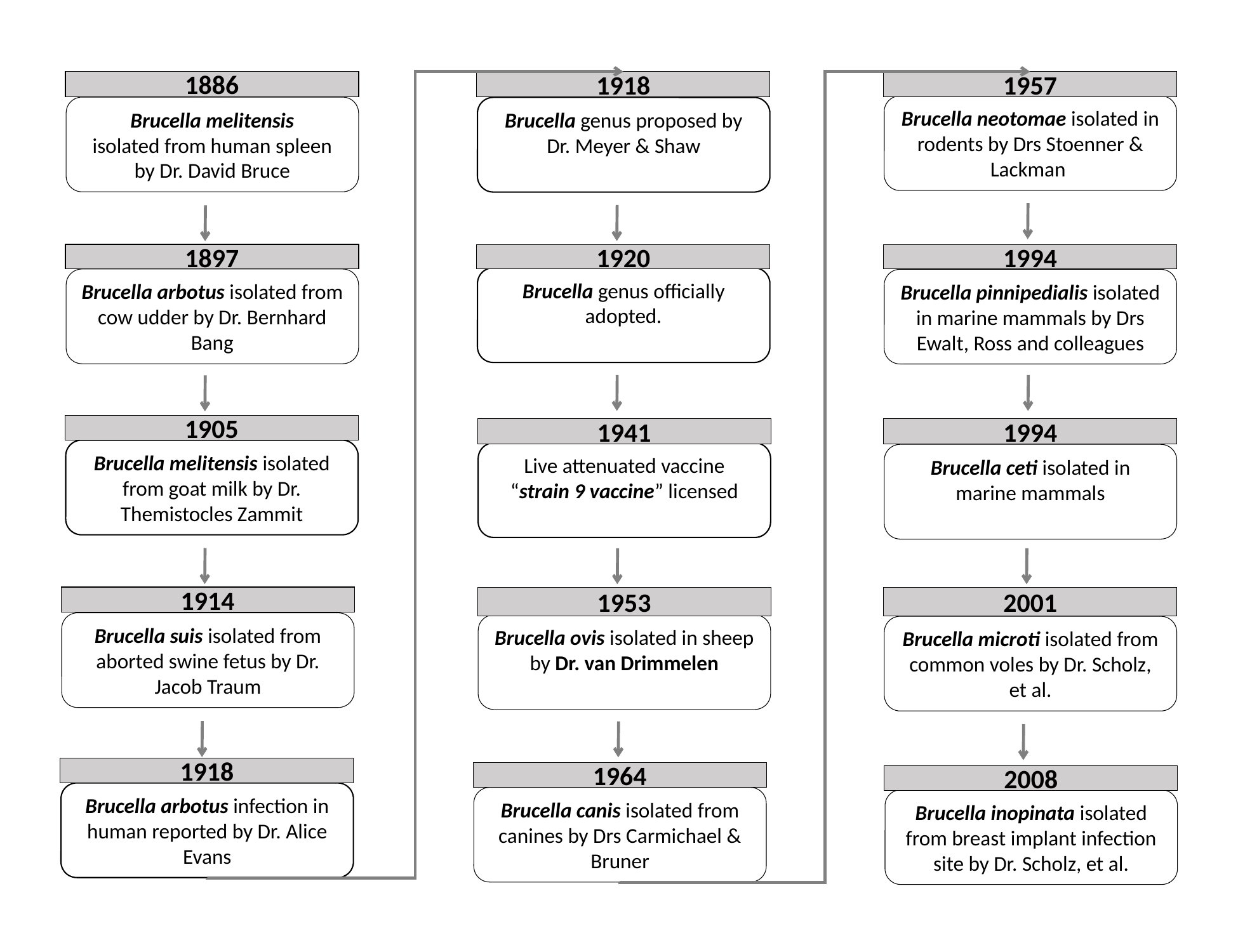

1886
1957
1918
Brucella neotomae isolated in rodents by Drs Stoenner & Lackman
Brucella melitensis
isolated from human spleen by Dr. David Bruce
Brucella genus proposed by Dr. Meyer & Shaw
1897
1920
1994
Brucella genus officially adopted.
Brucella arbotus isolated from cow udder by Dr. Bernhard Bang
Brucella pinnipedialis isolated in marine mammals by Drs Ewalt, Ross and colleagues
1905
1941
1994
Brucella melitensis isolated from goat milk by Dr. Themistocles Zammit
Live attenuated vaccine “strain 9 vaccine” licensed
Brucella ceti isolated in marine mammals
1914
1953
2001
Brucella suis isolated from aborted swine fetus by Dr. Jacob Traum
Brucella ovis isolated in sheep by Dr. van Drimmelen
Brucella microti isolated from common voles by Dr. Scholz, et al.
1918
1964
2008
Brucella arbotus infection in human reported by Dr. Alice Evans
Brucella canis isolated from canines by Drs Carmichael & Bruner
Brucella inopinata isolated from breast implant infection site by Dr. Scholz, et al.
