# Supplementary material for: ERAIZDA: a model for holistic annotation of animal infectious and zoonotic diseases
Source: Database (Oxford). 2015 Nov 18;2015:bav110. doi: 10.1093/database/bav110 (PMC4651161; doi:10.1093/database/bav110)
Supplement: Supplementary Data [file supp_bav110_suppl_data.zip › SupplemetaryFile_6.pptx]

## Slide 1
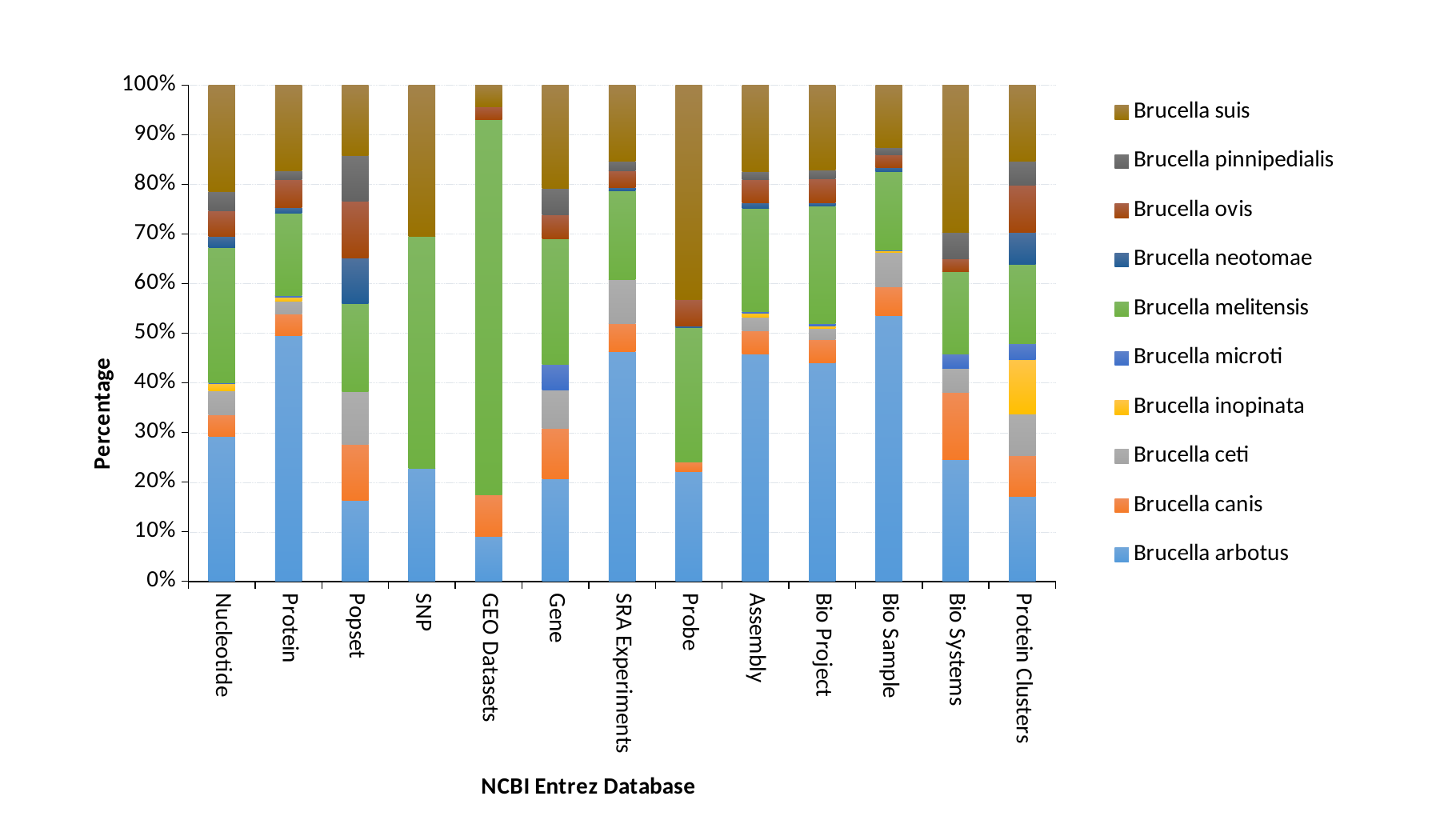

### Chart
| Category | Brucella arbotus | Brucella canis | Brucella ceti | Brucella inopinata | Brucella microti | Brucella melitensis | Brucella neotomae | Brucella ovis | Brucella pinnipedialis | Brucella suis |
|---|---|---|---|---|---|---|---|---|---|---|
| Nucleotide | 7068.0 | 1049.0 | 1181.0 | 349.0 | 45.0 | 6591.0 | 529.0 | 1250.0 | 949.0 | 5176.0 |
| Protein | 470202.0 | 41635.0 | 24289.0 | 7676.0 | 3879.0 | 156828.0 | 10429.0 | 53730.0 | 16886.0 | 164072.0 |
| Popset | 23.0 | 16.0 | 15.0 | None | None | 25.0 | 13.0 | 16.0 | 13.0 | 20.0 |
| SNP | 9961.0 | None | None | None | None | 20536.0 | None | None | None | 13340.0 |
| GEO Datasets | 17.0 | 16.0 | None | None | None | 142.0 | None | 5.0 | None | 8.0 |
| Gene | 13721.0 | 6763.0 | 5159.0 | None | 3412.0 | 16756.0 | None | 3202.0 | 3504.0 | 13804.0 |
| SRA Experiments | 275.0 | 33.0 | 53.0 | None | None | 106.0 | 4.0 | 20.0 | 11.0 | 91.0 |
| Probe | 109.0 | 10.0 | None | None | None | 133.0 | 2.0 | 26.0 | None | 213.0 |
| Assembly | 155.0 | 16.0 | 9.0 | 3.0 | 1.0 | 70.0 | 4.0 | 16.0 | 5.0 | 59.0 |
| Bio Project | 314.0 | 33.0 | 16.0 | 3.0 | 4.0 | 168.0 | 5.0 | 34.0 | 13.0 | 121.0 |
| Bio Sample | 433.0 | 47.0 | 56.0 | 2.0 | 2.0 | 127.0 | 7.0 | 21.0 | 11.0 | 102.0 |
| Bio Systems | 2002.0 | 1105.0 | 399.0 | None | 231.0 | 1348.0 | None | 213.0 | 441.0 | 2412.0 |
| Protein Clusters | 3329.0 | 1577.0 | 1628.0 | 2099.0 | 646.0 | 3077.0 | 1241.0 | 1836.0 | 942.0 | 2959.0 |
