# Supplementary material for: ERAIZDA: a model for holistic annotation of animal infectious and zoonotic diseases
Source: Database (Oxford). 2015 Nov 18;2015:bav110. doi: 10.1093/database/bav110 (PMC4651161; doi:10.1093/database/bav110)
Supplement: Supplementary Data [file supp_bav110_suppl_data.zip › SupplemetaryFile_7.docx]

**Supplementary File 7. Example of brucellosis disease biomarker manually annotated from PubMed articles**

| **Biomarker Name** |  | **Symbol** | **Group** | **Class** | **Experimental species** | **Evidence (Pubmed ID)** | **Date** | **Supporting Text** |
| --- | --- | --- | --- | --- | --- | --- | --- | --- |
| Zinc-dependent metallopeptidase |  | BAB1_0270 | uncharacterized protein | virulence factor | Brucella abortus (strain 2308) | 24928771 | 2014 | The mutants (∆0267 & ∆0270) efficiently invaded HeLa and J774.A1 cells but both mutants showed a decreased intracellular survival in macrophages and HeLa cells 72 and 96 h post-infection, respectively, and were non-detected in J774.A1 cells 120 h post infection. With respect to in vivo persistence ∆0267 was detected through the fourth week while ∆0270 decreased at 7 days disappearing the second week. BAB1_0267 and BAB1_270 are necessary to establish an optimal infectious process in B. abortus 2308, |
| Hypothetical protein |  | BAB1_0267 | uncharacterized protein | virulence factor | Brucella abortus (strain 2308) | 24928771 | 2014 | The mutants (∆0267 & ∆0270) efficiently invaded HeLa and J774.A1 cells but both mutants showed a decreased intracellular survival in macrophages and HeLa cells 72 and 96 h post-infection, respectively, and were non-detected in J774.A1 cells 120 h post infection. With respect to in vivo persistence ∆0267 was detected through the fourth week while ∆0270 decreased at 7 days disappearing the second week. BAB1_0267 and BAB1_270 are necessary to establish an optimal infectious process in B. abortus 2308, |
| Nucleoside diphosphate kinase |  | NDP | enzyme | vaccine candidate | Brucella abortus (strain 544) | 25724777 | 2015 | Recombinant Nucleoside diphosphate kinase (rNDP) immunization enables to elicit both of the humoral and cellular response, ultimately enhancing protection level in experimental mice, suggesting that rNdk of B. abortus might be a useful candidate for subunit vaccine for brucellosis in animals.s |
| Phosphoribosylamine--glycine ligase |  | purD | enzyme | mutant | Brucella abortus RB51-AHVLA | 25546140 | 2015 | Mutation of purD and purF genes attenuates Brucella abortus strain RB51. Immunization with purD and purF mutants protected mice against a challenge with the virulent B. abortus strain 544. Genes encoding the purD and purF are required for intracellular survival and virulence of B. abortus. |
| Amidophosphoribosyltransferase |  | purF | enzyme | mutant | Brucella abortus RB51-AHVLA | 25546140 | 2015 | Mutation of purD and purF genes attenuates Brucella abortus strain RB51. Immunization with purD and purF mutants protected mice against a challenge with the virulent B. abortus strain 544. Genes encoding the purD and purF are required for intracellular survival and virulence of B. abortus. |
| ATP-binding/permease protein |  | cydC | protein motif | mutant | Brucella abortus biovar 1 strain IVKB9007 | 25253663 | 2014 | The cydC & looP mutants were found to be virtually incapable of intracellular replication in both murine macrophages (RAW264.7) and the HeLa cell line, and their virulence was significantly impaired in BALB/c mice. Intraperitoneal immunization of mice with a dose of the live IVKB9007 looP::Tn5 and IVKB9007 cydC::Tn5 mutants provided a high degree of protection against challenge with pathogenic B. abortus strain 544. |
| ATP/GDP-binding protein |  | looP | protein | mutant | Brucella abortus biovar 1 strain IVKB9007 | 25253663 | 2014 | The cydC & looP mutants were found to be virtually incapable of intracellular replication in both murine macrophages (RAW264.7) and the HeLa cell line, and their virulence was significantly impaired in BALB/c mice.Intraperitoneal immunization of mice with a dose of the live IVKB9007 looP::Tn5 and IVKB9007 cydC::Tn5 mutants provided a high degree of protection against challenge with pathogenic B. abortus strain 544. |
| Ribosomal protein L9 |  | L9 | protein | vaccine candidate | Brucella abortus (strain 544) | 23913725 | 2014 | Ribosomal protein L9 produced as a recombinant protein and studied in mouse model for vaccine potential was found to be immunogenic in terms of generating serum antibody response and release of IFN-y from mice spleen cells. Recombinant L9-immunized mice were protected against challenge with virulent B. abortus strain 544 |
| mir-1981 |  | mir-1981 | microRNA | gene regulator | Mouse RAW264.7 cells | 22904669 | 2012 | The interactions of miR-1981 (expressed in RAW264.7 cells) and its target genes, Bcl-2 were validated by luciferase assay and the results showed that miR-1981 mimic up-regulated the luciferase activity of psiCHECK-2 Bcl-2 3' UTR. The results provide valuable framework on Brucella induced miRNA, and suggest that Brucella may establish chronic infection by regulating miRNA expression profile. |
| Histidinol dehydrogenase |  | hisD | enzyme | drug target | Brucella suis biovar 1 (strain 1330) | 17481905 | 2007 | Series of substituted benzylic ketones derived from histidine inhibited Brucella suis Histidinol dehydrogenase (HDH) in the lower nanomolar range and constitute attractive candidates for the development of novel anti-Brucella agents. |
| Histidinol dehydrogenase |  | hisD | enzyme | drug target | Brucella suis biovar 1 (strain 1330) | 17698620 | 2007 | Series of substituted benzylic ketones derived from histidine inhibited Brucella suis Histidinol dehydrogenase (HDH) in the lower nanomolar range and constitute attractive candidates for the development of novel anti-Brucella agents. |
| Beta-carbonic anhydrase |  | bsCA 1 | enzyme | drug target | Brucella suis | 20211561 | 2010 | The best bsCA 1 inhibitor were the galactose and ribose sulfanilamides, with inhibition constants of 8.9-9.2 nM |
| Beta-carbonic anhydrase |  | bsCA II | enzyme | drug target | Brucella suis | 21251841 | 2011 | The best bsCA II inhibitors were some glycosylated sulfanilamides, aliphatic sulfamates, and halogenated sulfanilamides, with inhibition constants of 7.3-87nM. |
