# Supplementary material for: ERAIZDA: a model for holistic annotation of animal infectious and zoonotic diseases
Source: Database (Oxford). 2015 Nov 18;2015:bav110. doi: 10.1093/database/bav110 (PMC4651161; doi:10.1093/database/bav110)
Supplement: Supplementary Data [file supp_bav110_suppl_data.zip › SupplementaryFile1.docx]

**Supplementary File 1. Top 100 Google hits of search terms (brucellosis OR (brucellosis disease) OR (brucellosis zoonotic disease) OR (brucellosis in animal) OR (brucellosis in human)) as of 04/28/2015**

| **Google Hit (Unfiltered)** | **Source** | **Description** | **PADER** |
| --- | --- | --- | --- |
| 1. WHO \| Brucellosis | WHO | World Health Organization | A |
| 2. [PDF]Brucellosis (human) - World Health Organization | WHO | World Health Organization | A |
| 3. Brucellosis in Humans | VetMed | Veterinary Medicine - University affiliated | E |
| 4. Brucellosis – Regionally Emerging Zoonotic Disease? | NCBI-PMC | NCBI Pubmed Central | P |
| 5. Zoonotic - Brucellosis - Infectious Disease Epidemiology ... | MCDC | Maine Center for Disease Control and Prevention | E |
| 6. Zoonotic Diseases:Disease Transmitted from Animals to ... | DH | Minnesota Department of Health | E |
| 7. [PDF]Pathogenesis and pathobiology of zoonotic brucellosis ... - OIE | OIE | World Organisation for Animal Health | A |
| 8. [PDF]Brucella - OIE | OIE | World Organisation for Animal Health | A |
| 9. CDC - Home - Brucellosis | CDC | Center for Disease Control and Prevention | A |
| 10. Brucellosis \| Florida Department of Health | DH | Florida Department of Health | E |
| 11. Brucellosis - Medscape Reference | eMedicine | Medscape Medical News | E |
| 12. [PDF]Brucella canis Infections in Humans - NASPHV | NASPHV | National Association of State Public Health Veterinarians | E |
| 13. Brucellosis « CFSPH | CFSPH | Center for Food Security and Public Health | A |
| 14. Zoonotic Diseases \| Georgia Department of Public Health | DH | Georgia Department of Public Health | E |
| 15. OMICS Group : eBooks :: Brucellosis: A Global Re-emerging ... | eBOOK | OMICS Group International – eBooks | P |
| 16. Retrospective and prospective perspectives on zoonotic ... | Article | Journal: Frontiers in Microbiology | P |
| 17. [PDF]Foreign Animal and Zoonotic Disease Information for Small ... | University | Texas A&M University | E |
| 18. Brucellosis \| AKC Canine Health Foundation | CHF | Canine Health Foundation | A |
| 19. Zoonotic Diseases of Cattle \| Publications and Educational ... | University | Virginia Polytechnic Institute and State University | E |
| 20. [PDF]Brucellosis: A re-emerging zoonosis Veterinary Microbiology | Researchgate | Social networking site for scientists and researchers | E |
| 21. Zoophilia and health - Wikipedia, the free encyclopedia | Wikipedia | An open-access encyclopedia | E |
| 22. [PDF]Human Brucellosis - The Journal of the American Board of ... | Article | Journal: American Board of Family Medicine | P |
| 23. Brucellosis in Humans and Animals - Page 13 - Google Books Result | BOOK | Google books | P |
| 24. Veterinary Public Health Association - Zoonoses - Brucellosis | Association | Veterinary Public Health Association, India | E |
| 25. Marine mammal brucellosis: a new dimension to an old ... | Article | Journal: Current Science | P |
| 26. TAHC — Bovine Brucellosis Page | TAHC | Texas Animal Health Commission | E |
| 27. Brucellosis Prevention - eMedicineHealth | eMedicine | Medscape Medical News | E |
| 28. [PDF]National Brucellosis Surveillance Plan - Draft - APHIS - US ... | APHIS | USDA Animal and Plant Health Inspection Service | A |
| 29. Essentials of Rubin's Pathology - Page 221 - Google Books Result | BOOK | Google Books | P |
| 30. Rubin's Pathology: Clinicopathologic Foundations of Medicine | BOOK | Google Books | P |
| 31. One Health: The Theory and Practice of Integrated Health ... | BOOK | Google Books | P |
| 32. Brucellosis in children: Prevention, diagnosis and ... | Article | ScienceDirect | P |
| 33. [PDF]Seroprevalence of brucellosis in animals and human ... | Article | Journal: Eurosurveillance | P |
| 34. Global Burden of Human Brucellosis: A Systematic Review ... | Article | Journal: PLOS Neglected Tropucal Diseases | P |
| 35. Feral Hogs and Disease « Texas Natural Wildlife - AgriLife.org | Article | Texas A&M AgriLife | P |
| 36. Prevalence of Brucellosis among Women Presenting with ... | Article | Journal: Hindawi | P |
| 37. the Brucella Bioinformatics Portal (BBP)! - Welcome to ... | PHIDIAS-BBP | Pathogen-Host Interaction Data Integration and Analysis System: Brucella Bioinformatics Portal | D |
| 38. Pakistan: Crimean Congo haemorrhagic fever and ... - Hubnet | Hubnet | One Health Network South Asia | E |
| 39. Increasing awareness of zoonotic diseases among health ... | BOOK | Google Books | P |
| 40. Zoonoses and Communicable Diseases Common to Man and ... | BOOK | Google Books | P |
| 41. [PDF]Zoonosis Update - American Veterinary Medical Association | AVMA | American Veterinary Medical Association | E |
| 42. Oxford Textbook of Medicine | BOOK | Google Books | P |
| 43. Brucellosis in Pregnant Women - Clinical Infectious Diseases | Article | Journal: Clinical Infectous Diseases | P |
| 44. Madkour’s Brucellosis - Google Books Result | BOOK | Google Books | P |
| 45. Fact Sheet - Brucellosis - Animals - Canadian Food ... | CFIA | Canadian Food Inspection Agency | A |
| 46. One Health units and brucellosis in Kenya - SlideShare | Slideshare | Online Slide Share Network | E |
| 47. [PDF]Epidemiologic Summary of Human Brucellosis in California ... | DH | California Department of Public Health | E |
| 48. BMC Infectious Diseases \| Full text \| Human brucellosis ... | Article | Journal: BMC Infectious Diseases | P |
| 49. Brucellosis \| Doctor \| Patient.co.uk | Patient | Patient UK | E |
| 50. [PDF]Autoimmune Changes in Human - International Journal of ... | Article | Journal:International Journal of Biopharmaceutics | P |
| 51. Current Methods of Human and Animal Brucellosis ... | Article | Journal: Advances in Infectious Diseases | P |
| 52. Diagnostic lab keeps dogs healthy with new test for canine ... | University | Kansas State University | E |
| 53. Oxford Textbook of Zoonoses: Biology, Clinical Practice, ... | BOOK | Google Books | P |
| 54. brucellosis - definition of brucellosis by The Free Dictionary | Dictionary | The free Dictionary | E |
| 55. Animal Science Reviews 2010 - Page 172 - Google Books Result | BOOK | Google Books | P |
| 56. Clinical manifestations, diagnosis, and treatment of brucellosis | UpToDate | Wolters Kluwer Health | E |
| 57. Quinolones for Treatment of Human Brucellosis: Critical ... | Article | Journal: Antimicrobial Agents and Chemotherapy | P |
| 58. Brucellosis: Endemic and re-emerging zoonosis \| Infectious ... | News | Healio: Infectious Disease News | R |
| 59. Spatial analysis on human brucellosis incidence in ... | Article | Journal: BMJ Open | P |
| 60. [PDF]Brucellosis Infection Control - Communicable Disease ... | DH | San Francisco Department of Public Health | R |
| 61. CAPACITY BUILDINGFOR SURVEILLANCE AND ... | FAO | Food and Agriculture Organisation | A |
| 62. One Health Activities \| ZDU – Republic of Kenya Zoonotic ... | ZDU | Kenya Zoonotic Disease Unit | E |
| 63. New vaccines against an old disease: brucellosis \| Science ... | News | New vaccines against an old disease: brucellosis | R |
| 64. Brucellosis - PA.us | PGC | Pennsylvania Game Commission | A |
| 65. Human brucellosis among pyrexia of unknown origin cases ... | Article | Journal: Emerging Health Threats | P |
| 66. Brucellosis - An Infectious Re-Emerging Bacterial Zoonosis ... | Article | JournalL International Journal of Livestock Research | P |
| 67. [PDF]OCCUPATIONAL BRUCELLOSIS AMONG HIGH RISKY ... | Article | Journal: The Medical Journal of Basrah University | P |
| 68. Investigating the impact of brucellosis on public ... - R4D | R4D | Research 4 Development, UKAID | R |
| 69. [PDF]Brucellosis in Sheep and Goats - European Commission | EC | Eurprean Commision | R |
| 70. [PDF]Human Brucellosis Risk From Feral Swine - www.UAEX.edu. | University | University of Arkansas | E |
| 71. Going out with a Bangs: Control of Human Brucellosis by ... | Blogs | ScienceBlogs | R |
| 72. Time-Series Analysis on Human Brucellosis During 2004 ... | Article | Journal: Zoonoses and Public Health | P |
| 73. [PDF]Review of clinical and laboratory features of human ... | Article | Journal: Indian Journal of Medical Micribiology | P |
| 74. Zoonotic diseases, A-Z - King County | DH | Public Health - Seattle & King County | E |
| 75. Brucella group Database - Broad Institute | BGD | Brucella Group Database | D |
| 76. The new global map of human brucellosis - The Lancet | Article | Journal: The Lancet Infectious Diseases | P |
| 77. Concurrent Brucellosis and Q Fever Infection: a Case ... | Article | Journal: Central Asian Journal of Global Health | P |
| 78. NC DPH: Brucellosis - Epi | DH | North carolina Department of Health and Human Services | E |
| 79. Human brucellosis occurrences in inner mongolia, China: a ... | Article | Journal: BMC Infectious Diseases | P |
| 80. APHIS Eradication of Brucellosis in Wildlife - EMWH.org | APHIS | USDA Animal and Plant Health Inspection Service | A |
| 81. [PDF]brucellosis - Louisiana Department of Health and Hospitals | DH | Louisiana Department of Health and Hospitals | E |
| 82. [PDF]a study on the seroprevalence of brucellosis in human and | Article | Journal of Animal and Plant Sciences | P |
| 83. [PDF]Zoonotic disease risk - feral pigs - NSW Department of ... | ABU | Animal BiosecurityUnit, Department of Trade and Investment, Regional Infrastructure and Services | E |
| 84. Bulletin of the World Health Organization - Human health ... | WHO | World Health Organization | A |
| 85. Molecular epidemiology of brucellosis in northern Tanzania | University | University of Glasgow | E |
| 86. www.infonet-biovision.org - Brucellosis | InfoBiovision | Infonet-Biovision-Animal Diseases | E |
| 87. [PPT]BRUCELLOSIS SITUATION IN KENYA - Wyoming ... | Presentation | University of Wyoming | E |
| 88. [PDF]Brucellosis: Understanding an Important Arctic Infectious ... | CCH | Center for Climate and Health, Alaska | R |
| 89. [PDF]Recent lessons learnt from the outbreak of brucellosis in ... | Article | Journal: Journal of Eritrean Medical Association | P |
| 90. [PDF]guidelines for the diagnosis, management - Kementerian ... | DH | Disease Control Division, Department of Public Health, Ministry of Health Malaysia | E |
| 91. Brucellosis - Factsheet - Health Protection Surveillance Centre | HPSC | Health Protection Surveillance Centre | A |
| 92. Zoonoses: Relieving the burden of brucellosis? | News | Agriculturist | R |
| 93. Brucellosis in Africa \| ADVANZ | ADVANZ | Advocacy for Neglected Zoonotic Diseases | D |
| 94. Fulltext \| Prevalence study of brucellosis among high-risk ... | Article | Microbiology Discovery, Herbert Open Access Journal | P |
| 95. Human Brucellosis in Traditional Pastoral Communities in ... | Article | International Journal of Tropical Medicine | P |
| 96. [PDF]Brucellosis - Arizona Department of Health Services | DH | Arizona Department of Health Services | E |
| 97. [PDF]Brucellosis Investigation Guideline - Kansas Department of ... | DH | Kansas Department of Health | E |
| 98. Severe Thrombocytopenia In Acute Brucellosis: A Case ... | Article | Journal: Internet Scientific Publications | P |
| 99. [PDF]Brucellosis and Tuberculosis in Arsi-Negele District ... | Article | Journal: Tropicultura | P |
| 100. Student's yak research may reduce infection in Nepal ... | News | Cornell Chronicle | R |
