# Supplementary material for: ERAIZDA: a model for holistic annotation of animal infectious and zoonotic diseases
Source: Database (Oxford). 2015 Nov 18;2015:bav110. doi: 10.1093/database/bav110 (PMC4651161; doi:10.1093/database/bav110)
Supplement: Supplementary Data [file supp_bav110_suppl_data.zip › SupplementaryFile2.docx]

**Supplementary File 2. Detailed description of ERAIZDA annotation parameters**

1. **Disease parameters**

*Nomenclature of infectious diseases*

Common disease names are used, but there are some cases where same disease is identified with different names due to a lack of standardized nomenclature. In the *ERAIZDA* model each recognized disease name will be annotated as a separate disease and will be associated with a Disease Ontology (DO) [1, 2] standardized term (if available). Use of DO terms facilitates disease knowledge exchange across related biomedical domains such as the Open Biological and Biomedical Ontologies (OBO) [3].

*Classification of diseases*

The model adopts different terminologies for describing *infection*, *frequency*, and *typology* of an infectious or zoonotic disease (Table 1). The disease typology was first defined by the Commission on Macroeconomics and Health (CMH) [4] and later was accepted by the WHO Consultative Expert Working Group on Research and Development (R&D), Financing and Coordination (CEWG) [5]. Knowing the typology could guide in monitoring of disease burden in high and low income countries.

*Specific features of diseases*

The *signs* and *symptoms* are major factors to be considered in disease annotation. Symptoms are more subjective and can simplify the clinical diagnosis process especially to rule out unrelated diseases. Unfortunately, some symptoms are clinically indistinguishable but detailed description of all observed signs should help in characterizing a disease. The *ERAIZDA* model expects all signs and symptoms of each represented disease to be annotated in detail from all available diverse *PADER* sources to facilitate better understanding of the underlying ailment. Whenever available Other important annotation parameters to be recorded include the *outbreaks*, the *distribution* and the *case fatality rate* (CFR) of a disease. Outbreaks in multiple years could signify reemerging disease while outbreaks in recent years may signify an emerging disease. Disease *distribution* can assist in determining *frequency* and *typology* described in Table 1. Associating CFR with a disease is important for measuring the severity of a particular disease and thus improve decision making. Unexpected higher CFR should increase suspicion of an emerging or foreign disease. To reduce the CFR it is important to also record the associated *risk factors* which include the controllable and uncontrollable dynamics that can increase risk of contracting infectious diseases. Also considered in the *ERAIZDA* model are clinical parameters including *diagnosis*, *treatment* and *preventive* measures reported in the *PADER*. The use of pathology in disease investigation will be considered. In this case, the macroscopic and microscopic lesions will be indicated as clinical signs and symptoms.

1. **Pathogen parameters**

*Classification of causal agents*

The type of *pathogen* that causes infectious diseases is a basic parameter for better understanding of disease management. The most well-known pathogenic agents that can cause infections in animals and human are viruses [6-8], bacteria [9, 10], protozoa [11-13], fungi [14], rickettsia [15], metazoa [16, 17], and prions [18]. In the ERAIZDA model rickettsia are considered a separate group of bacteria because they have the common feature of being spread by arthropod vectors i.e. lice, fleas, mites and ticks [15, 19]. Once the causal agent is identified it is important to know its taxonomy for effective disease management. The most important level of taxonomic classification of any pathogenic agent is the species. Classification below species is very crucial for characterizing a disease outbreak, especially in differentiating reemerging from emerging pathogens and for making better decisions for developing strain-specific vaccines or therapeutic measures. The NCBI Taxonomy Group has been assigning unique taxon identifiers down to strain-level to facilitate identification and characterization, but due to significant growth in genome sequencing, this effort ceased for microbial genomes submitted beyond January 2014 [20]. Whenever available strain information will be annotated and included under the subspecies parameter.

*Specific features of infectious agents*

Different pathogenic agents exhibit diverse characteristics that provide a better understanding of why some diseases pose a greater threat to some hosts than others. The following characteristics, with definitions adopted from medical and veterinary dictionaries [21], are deliberately included in the ERAIZDA model to provide clarity and improve understanding of the infectious agent. These include: (i) *infectivity* - the ability of the agent to enter a host and produce infection; (ii) *pathogenicity* - the ability of the agent to cause disease in an infected host; (iii) *virulence* - the severity of the infection caused by pathogenic agent; (iv) *toxigenicity* - the ability of the agent to produce a toxin that causes disease; (v) *antimicrobial resistance* - the ability of microbes to grow in the presence of a chemical (drug) that would normally kill them or limit their growth; and (vi) *antigenicity* - the ability of the agent to induce antibody production in the host.

Another important specific parameter is the *reservoir* where the infectious agent normally resides, grows, and multiplies. A disease agent is capable of living in multiple habitats such as soil, water, air, wood, animal body, food, litter, manure, etc. However, for an infection to occur the infectious agent must leave the reservoir through various ways commonly, referred to as *portals of exit* and enter a *susceptible host* by either direct *transmission* or indirect transmission [22]. The *incubation* period, the period taken by an infectious agent to reach a threshold necessary to produce symptoms in the infected host is key in understanding mechanism of infection at the host-pathogen interaction level. The above described pathogen features are important parameters that can be annotated in detail from PADER sources. This list of parameters may not be complete but with better knowledge additional parameters might be added in the future.

1. **Molecular Biomarkers**

Knowledge of molecular *biomarkers* related to animal infectious and zoonotic diseases is crucial for better understanding of diagnosis, prognosis, and control of infectious agents and in detecting any emerging drug resistance. Specific disease related biomarkers i.e. proteins, microRNAs, and/or single nucleotide polymorphisms (SNPs) are very important in understanding factors that instigate disease development. Also considered as biomarkers are the molecules that interact with specific drugs (*drug targets*) that are administered to treat or diagnose infectious diseases. Example includes specific enzymes, ion channels, nuclear receptors and G protein-coupled receptors (GPCRs). The GPCRs form nearly 50% of the known drug targets [21]. Other type of molecular biomarkers are the *toxins* produced by the causal agents, for example, *anthrax toxin* is produced by *Bacillus anthracis*, a bacterium that causes anthrax. The molecular components of anthrax toxin are three different toxic proteins, the *protective antigen* (PA), *lethal factor* (LF), and *edema factor* (EF) [23]. Expression of genes that code for microbial toxic proteins could suggest infection likelihood. We believe that through manual annotation of disease-specific publications it is possible to identify a significant number of experimentally validated disease-specific molecular biomarkers.

**Summary**

Table 1 below summarizes the ERAIZDA annotation parameters. For clarification each parameter is assigned a name, description, a value and example obtained from the preliminary annotation of brucellosis.

Table 1. Summary of ERAIZDA annotation parameters for animal infectious and zoonotic diseases

| **Parameter** | **Brief Description** | | **Value** | **Example** |
| --- | --- | --- | --- | --- |
| ***A: Disease parameters*** | | | | |
| 1. Disease Name | Disease name as listed in popular health agencies such as OIE, WHO, CDC, etc. | | *Common Name* | Bovine Brucellosis |
| 1. Disease Name synonyms | Other names commonly used. For zoonotic diseases some names may apply only in animals, others may only apply to infections in human | | *Synonyms* | Malta fever, Undulant fever, Mediterranean fever, Bang’s fever |
| 1. Ontology (DO) name | Disease Ontology (DO) [1, 2] term of the common disease is used as a standard name to facilitate cross-referencing to other major biomedical health ontologies [3]. | | *DO name* | *Brucella abortus* brucellosis |
| 1. DO Identifier | This is a unique identifier of the DO name. | | *DO Identifier* | DOID: 14457 |
| 1. Listing Agency | The agencies that recognize the disease. If not listed may signify emerging or neglected disease. | | *Name of agency* | OIE, WHO, CDC, FAO, GLEWS |
| 1. Causal agent | Category of the biological agents that can cause an infectious disease. These can be bacteria, virus, protozoa, parasite, prion, fungi, rickettsia or metazoa. | | *Name of the biological agent* | Bacteria |
| 1. Type of infection | An animal infectious disease can be transmitted from animal-to-animal (I), animal-to-human (Z), human-to-animal (Z), or human-to-human (I). | | *Infectious Disease (ID) or Infectious Zoonotic Disease (IZD) or Infectious with zoonotic potential (IZP)* | IZD |
| 1. Animal symptoms | General signs or symptoms observed in animals. These may include some clinical observations too. | | *Animal symptoms* | abortion, retained placenta, orchitis, epididymitis |
| 1. Human symptoms | General signs or symptoms observed in human. This applies for zoonotic infections of animal origin. These may include some clinical observations too. | | *Human symptoms* | abdominal pain, back pain, chills, excessive sweating, fatigue, undulant fever ,headache, joint pain, loss of appetite, weakness, weight loss, muscle pain, swollen glands |
| 1. Outbreaks | | Year of outbreak. Outbreaks in multiple years may signify reemergence of disease while outbreaks in recent years may signify an emerging disease. | *Year (newer), Year(old), etc.* | --- |
| 1. Distribution | | List of countries or regions where an outbreak has been reported. Similar regions as defined in OIE- World Animal Health Information Database (WAHID) be adopted. | *Country or Region* | Middle East, Asia, Africa, South America, Central America, Mediterranean Basin, Caribbean |
| 1. CMH Disease type | | The disease typology defined by the Commission on Macroeconomics and Health (CMH) [4], which was accepted by the WHO Consultative Expert Working Group on Research and Development (R&D): Financing and Coordination (CEWG) [24]. This data can facilitate informed R&D strategies especially monitoring of disease burden in high and low income countries. Type I: Diseases that occur in both rich and poor countries, with large numbers of vulnerable populations in each. Type II: Diseases that occur in both rich and poor countries, but with a substantial proportion of the cases in poor countries. Type III: Diseases that overwhelmingly or exclusively occur in developing countries. | *Type I, II or III* | Type II |
| 1. Disease frequency | | Disease frequency highlights the occurrence and distribution of infectious disease.  *Endemic:* A disease that occurs in a population with predictable regularity. The events are clustered in space but not in time.  *Sporadic:* A disease that is normally absent from a population but which can occur in that population.  *Epidemic/Epizootic:* A disease that occurs in a population in excess of its normally expected frequency of occurrence. The events are clustered in time and space.  *Pandemic:* A large epidemic affecting several countries or even one or more continents. | *Endemic, Sporadic, Epidemic, Pandemic* | Endemic |
| 1. Case Fatality Rate | | Case Fatality Rate (CFR) measures the severity of a disease, an important parameter to consider when annotating infectious diseases. Unexpected higher CFR should increase suspicion of something emerging or foreign. | *Percentage* | --- |
| 1. Risk factors | | These are variety of controllable and uncontrollable factors that can increase risk of contracting infectious diseases. | *---* |  |
| 1. Diagnosis | | Clinical diagnosis helps to rule out diseases that cause similar symptoms and to identify emerging, reemerging and foreign disease. | *culture, PCR, immuno-florescence & immuno-, chromatographic assays, etc.* | Screening, Rose Bengal test |
| 1. Treatment | | Therapy intended to cure the specified infectious disease or its cause. | *Known treatments* | Antibiotics |
| 1. Prevention | | Measures designed to prevent the introduction of a disease or spread of infection. | *Known preventive measures* | Vaccination |
| ***B: Pathogen parameters*** | | | | |
| 1. Family | | The family of the causal agent as represented in the NCBI taxonomy database [25]. | *Scientific name* | *Brucellaceae* |
| 1. Genus | | The genus of the causal agent as represented in the NCBI taxonomy database. | *Scientific name* | *Brucella* |
| 1. Species | | The species of the causal agent as represented in the NCBI taxonomy database. | *Scientific name* | *Brucella abortus* |
| 1. Species Taxons | | The total number of taxons classified under the species of the causal agent. | *NCBI statistics* | 170 |
| 1. Subspecies | | The subspecies of the causal agent as represented in the NCBI taxonomy database. | *Scientific name* | --- |
| 1. Subspecies Taxons | | The total number of taxons classified under the subspecies of the causal agent. | *NCBI statistics* | --- |
| 1. Taxon ID | | The unique taxon identifier assigned to the species or subspecies. | *Numerical Taxon ID* | 235 |
| 1. Taxon level | | This is a number that indicates the depth of classification (lineage) of the causal agent starting from species level. Zero is root level (species), one is the children of species (subspecies), three is the children of subspecies, and so forth. The higher the number the more specific the classification is. | *0, 1, 2, 3, etc.* | 0 |
| 1. Infectivity | | The ability of the causal agent to enter a host and produce infection. | *Low, Intermediate or High* | Intermediate |
| 1. Pathogenicity | | The ability of the causal agent to cause disease in an infected host. | *Low, Intermediate or High* | --- |
| 1. Virulence | | The severity of the infection. | *Low, Intermediate or High* | --- |
| 1. Toxigenicity | | The ability of the causal agent to produce toxin that causes disease. | *Low, Intermediate or High* | Low |
| 1. Resistance | | The ability of the causal agent to survive under adverse environmental conditions. | *Low, Intermediate or High* | --- |
| 1. Antigenicity | | The ability of the causal agent to induce antibody production in the host. | *Low, Intermediate or High* | --- |
| 1. Reservoir | | A habitat in which an infectious agent normally lives, grows, and multiplies. Reservoirs include humans, animals, and the environment. | *The actual name of reservoir e.g. soil, cattle, sheep, human, etc.* | wild reindeer, hares, bison, saiga antelopes |
| 1. Exit portal | | The path by which a pathogen leaves the reservoir or its host. | *Name of portal of exit e.g. urine, feces, secretion, eggs, milk, meat. etc.* | milk, uterine discharges, parturitions |
| 1. Entry portal | | A site through which an infectious agent enter the susceptible host and cause disease or infection. *Value:* | *Name of portal of entry e.g. mouth, skin, mucosal membrane, bites, etc.* | ingestion, mucous membranes, conjunctivae, wounds, skin |
| 1. Animal Hosts | | This is the name of any susceptible host. | *Animal name* | cattle, bison, buffalo, elk, yak, camels |
| 1. Human Host | | This applies for zoonotic diseases of animal origin. | *Yes or No* | Yes |
| 1. Transmission | | Infectious agents can be transmitted from infected organisms through various routes including direct or indirect contact with contaminated objects. *Value: Route or object name* | *Mode of transmission* | direct contact (with infected placenta, fetus, fetal fluids and vaginal discharges), ingestion (of unpasteurized milk, cheese and other dairy products) |
| 1. Incubation | | This is the time from exposure to an infectious agent until signs and symptoms of the disease appear. *Value: Number of days* | *Days, weeks, months, years?* | 2-4 weeks, months |
| 1. First Isolation | | Indicates the year the species was isolated and characterized. | *Year* | 1897 |
| ***C: Molecular parameters*** | | | | |
| 1. Biomarker Name | | This is an experimentally validated biomarker published in journal articles. Biomarkers can accelerate diagnosis, prognosis, characterization and therapy development processes. | *Standardized name e.g. Entrez name* | Ribosomal protein L9 |
| 1. Biomarker Symbol | | Biomarker approved symbol(s) | *Entrez Gene symbol* | *L9* |
| 1. Biomarker UniProtKB AC | | A UniProtKB accession is important to facilitate functional annotation of the gene-based biomarker. | *UniProtKB Accession* | N7T9T3 |
| 1. Biomarker Group | | This is the specific molecular group of the biomarker. *Value:* | *protein, microRNA, SNPs, transcript, etc.* | *Protein* |
| 1. Biomarker Class | | This defines the group of markers broadly. | *Example: vaccine candidate, virulence factor, toxin, drug target, mutant, antimicrobial, resistance, etc.* | Vaccine candidate |
| 1. Experimental Organism | | This is the experimental species or subspecies or model organism used in the investigation. | *Scientific name* | *Brucella abortus* (strain 544) |
| 1. Biomarker References | | All references supporting the biomarker | *Pubmed ID, Article DOI number, etc.* | PMID:23913725 |
| 1. Reference publication date | | Publication date of the reference. This is important for users to know how recent or how old the experiment is. | *Year* | 2014 |
| 1. Biomarker evidence Text | | Texts or sentences extracted from the supporting references. This is a short summary or conclusion that shows the validation of the biomarker. | *Text(s)* | Ribosomal protein L9 produced as a recombinant protein and studied in mouse model for vaccine potential was found to be immunogenic in terms of generating serum antibody response and release of IFN-y from mice spleen cells. Recombinant L9-immunized mice were protected against challenge with virulent B. abortus strain 544 |
| 1. URL for Molecular Data | | This is a site that shows most current molecular records of the infectious agent including nucleotides, proteins, genomes, assemblies, genes, SNPs, etc. These records give highlights of how well an infectious agent has been characterized and what biological projects have been supported thus facilitates informed research planning. | *The NCBI-Entrez record UR* | http://www.ncbi.nlm.nih.gov/Taxonomy/Browser/wwwtax.cgi?mode=Info&id=235&lvl=3&lin=f&keep=1&srchmode=1&unlock |

REFERENCES

1. Kibbe WA, Arze C, Felix V, Mitraka E, Bolton E, Fu G, Mungall CJ, Binder JX, Malone J, Vasant D *et al*: **Disease Ontology 2015 update: an expanded and updated database of human diseases for linking biomedical knowledge through disease data**. *Nucleic acids research* 2015, **43**(Database issue):D1071-1078.

2. Schriml LM, Arze C, Nadendla S, Chang YW, Mazaitis M, Felix V, Feng G, Kibbe WA: **Disease Ontology: a backbone for disease semantic integration**. *Nucleic acids research* 2012, **40**(Database issue):D940-946.

3. Smith B, Ashburner M, Rosse C, Bard J, Bug W, Ceusters W, Goldberg LJ, Eilbeck K, Ireland A, Mungall CJ *et al*: **The OBO Foundry: coordinated evolution of ontologies to support biomedical data integration**. *Nature biotechnology* 2007, **25**(11):1251-1255.

4. World Health Organization: **Report of the Commission on Macroeconomics and Health**. In: *Macroeconomics and Health: Investing in Health for Economic Development.* Edited by Steele H. WHO Library Cataloguing-in-Publication Data; 2001.

5. World Health Organization: **Defining Disease Types I, II and III**. In*.* Background document provided by the WHO Secretariat 14 November 2012.

6. Meng XJ: **Hepatitis E virus: animal reservoirs and zoonotic risk**. *Veterinary microbiology* 2010, **140**(3-4):256-265.

7. Walsh MG, Haseeb M: **The landscape configuration of zoonotic transmission of Ebola virus disease in West and Central Africa: interaction between population density and vegetation cover**. *PeerJ* 2015, **3**:e735.

8. Pigott DM, Golding N, Mylne A, Huang Z, Henry AJ, Weiss DJ, Brady OJ, Kraemer MU, Smith DL, Moyes CL *et al*: **Mapping the zoonotic niche of Ebola virus disease in Africa**. *eLife* 2014, **3**:e04395.

9. Leshem E, Meltzer E, Schwartz E: **Travel-associated zoonotic bacterial diseases**. *Current opinion in infectious diseases* 2011, **24**(5):457-463.

10. Higgins R: **Emerging or re-emerging bacterial zoonotic diseases: bartonellosis, leptospirosis, Lyme borreliosis, plague**. *Rev Sci Tech* 2004, **23**(2):569-581.

11. Fayer R, Dubey JP, Lindsay DS: **Zoonotic protozoa: from land to sea**. *Trends in parasitology* 2004, **20**(11):531-536.

12. Ouattara M, Silue KD, N'Guessan AN, Yapi A, Barbara M, Raso G, Utzinger J, N'Goran E: **Prevalence and polyparasitism of intestinal protozoa and spatial distribution of Entamoeba histolytica, E. dispar and Giardia intestinalis from pupils in the rural zone of Man in Cote d'Ivoire**. *Sante* 2008, **18**(4):215-222.

13. Gideon AA, Njine T, Nola M, Menbohan SF, Ndayo MW: **Measuring resistant forms of two pathogenic protozoa (Giardia spp and Cryptosporidium spp) in two aquatic biotopes in Yaounde (Cameroon)**. *Sante* 2007, **17**(3):167-172.

14. Sak B, Kvac M, Petrzelkova K, Kvetonova D, Pomajbikova K, Mulama M, Kiyang J, Modry D: **Diversity of microsporidia (Fungi: Microsporidia) among captive great apes in European zoos and African sanctuaries: evidence for zoonotic transmission?** *Folia parasitologica* 2011, **58**(2):81-86.

15. de Almeida RF, Garcia MV, Cunha RC, Matias J, e Silva EA, de Fatima Cepa Matos M, Andreotti R: **Ixodid fauna and zoonotic agents in ticks from dogs: first report of Rickettsia rickettsii in Rhipicephalus sanguineus in the state of Mato Grosso do Sul, mid-western Brazil**. *Experimental & applied acarology* 2013, **60**(1):63-72.

16. Mattiucci S, Garcia A, Cipriani P, Santos MN, Nascetti G, Cimmaruta R: **Metazoan parasite infection in the swordfish, Xiphias gladius, from the Mediterranean Sea and comparison with Atlantic populations: implications for its stock characterization**. *Parasite* 2014, **21**:35.

17. Spurgeon AN, Cress MC, Gabor O, Ding QQ, Tanaka T, Miller DC: **Oncogenic brain metazoan parasite infection**. *Case reports in neurological medicine* 2013, **2013**:263718.

18. Lee J, Kim SY, Hwang KJ, Ju YR, Woo HJ: **Prion diseases as transmissible zoonotic diseases**. *Osong public health and research perspectives* 2013, **4**(1):57-66.

19. Rozental T, Ferreira MS, Gomes R, Costa CM, Barbosa PR, Bezerra IO, Garcia MH, Oliveira ECDM, Galliez R, Oliveira S *et al*: **A cluster of Rickettsia rickettsii infection at an animal shelter in an urban area of Brazil**. *Epidemiol Infect* 2014:1-5.

20. Federhen S, Clark K, Barrett T, Parkinson H, Ostell J, Kodama Y, Mashima J, Nakamura Y, Cochrane G, Karsch-Mizrachi I: **Toward richer metadata for microbial sequences: replacing strain-level NCBI taxonomy taxids with BioProject, BioSample and Assembly records**. *Standards in genomic sciences* 2014, **9**(3):1275-1277.

21. Lundstrom K: **An overview on GPCRs and drug discovery: structure-based drug design and structural biology on GPCRs**. *Methods in molecular biology* 2009, **552**:51-66.

22. **Principles of Epidemiology in Public Health Practice, Third Edition: An Introduction to Applied Epidemiology and Biostatistics** [<http://www.cdc.gov/ophss/csels/dsepd/ss1978/lesson1/section10.html>]

23. Tama F, Ren G, Brooks CL, 3rd, Mitra AK: **Model of the toxic complex of anthrax: responsive conformational changes in both the lethal factor and the protective antigen heptamer**. *Protein Sci* 2006, **15**(9):2190-2200.

24. World Health Organization: **Defining Disease Types I, II and III.** In: *Background document provided by the WHO Secretariat 14 November 2012.*

25. Federhen S: **The NCBI Taxonomy database**. *Nucleic acids research* 2012, **40**(Database issue):D136-143.
