# Supplementary figures and images for: ERAIZDA: a model for holistic annotation of animal infectious and zoonotic diseases
Source: Database (Oxford). 2015 Nov 18;2015:bav110. doi: 10.1093/database/bav110 (PMC4651161; doi:10.1093/database/bav110)

## Slide 1
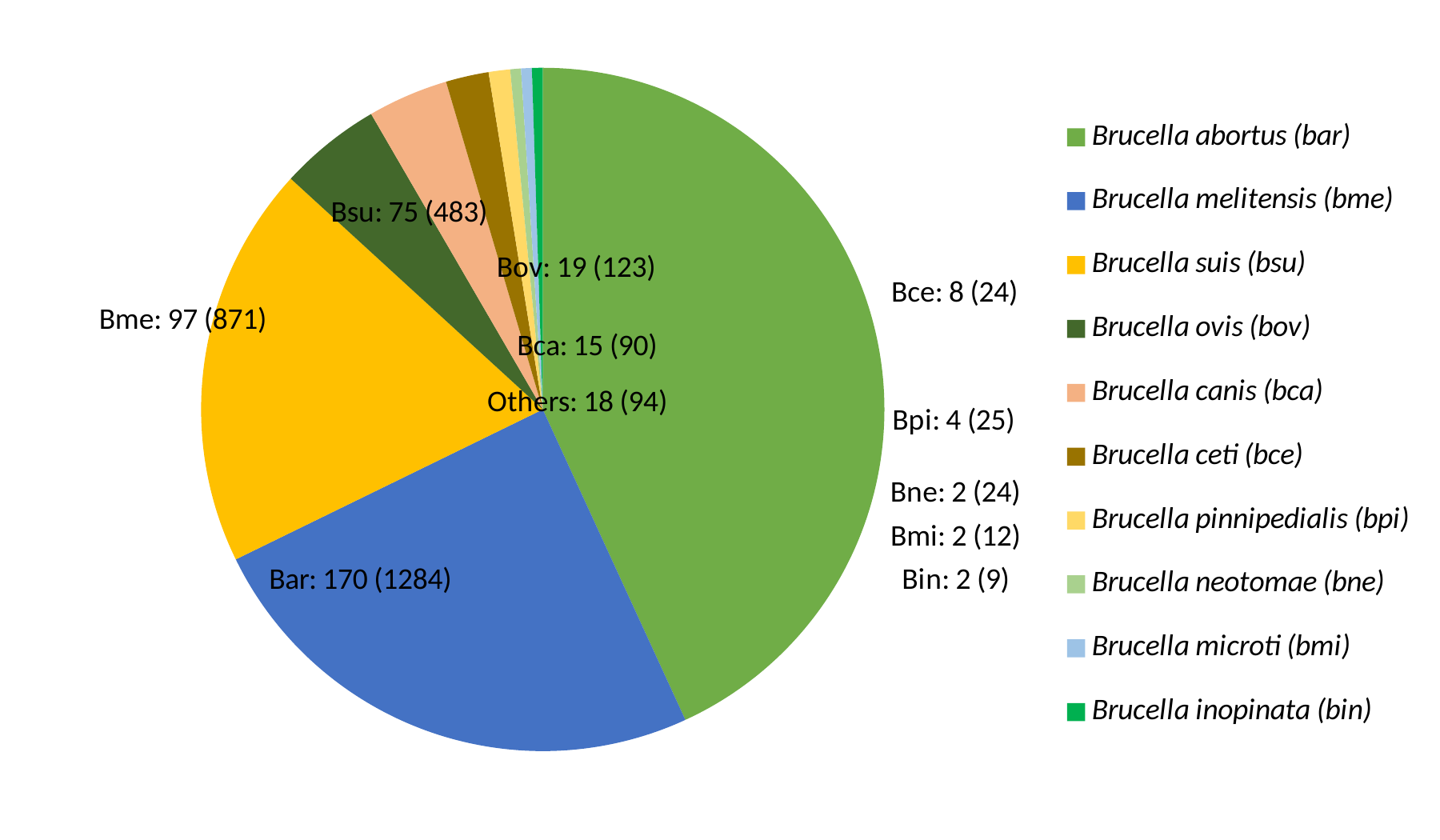

[unsupported chart]

Supplement: Supplementary Data [file supp_bav110_suppl_data.zip › SupplemetaryFile_5.pptx]

## Slide 1
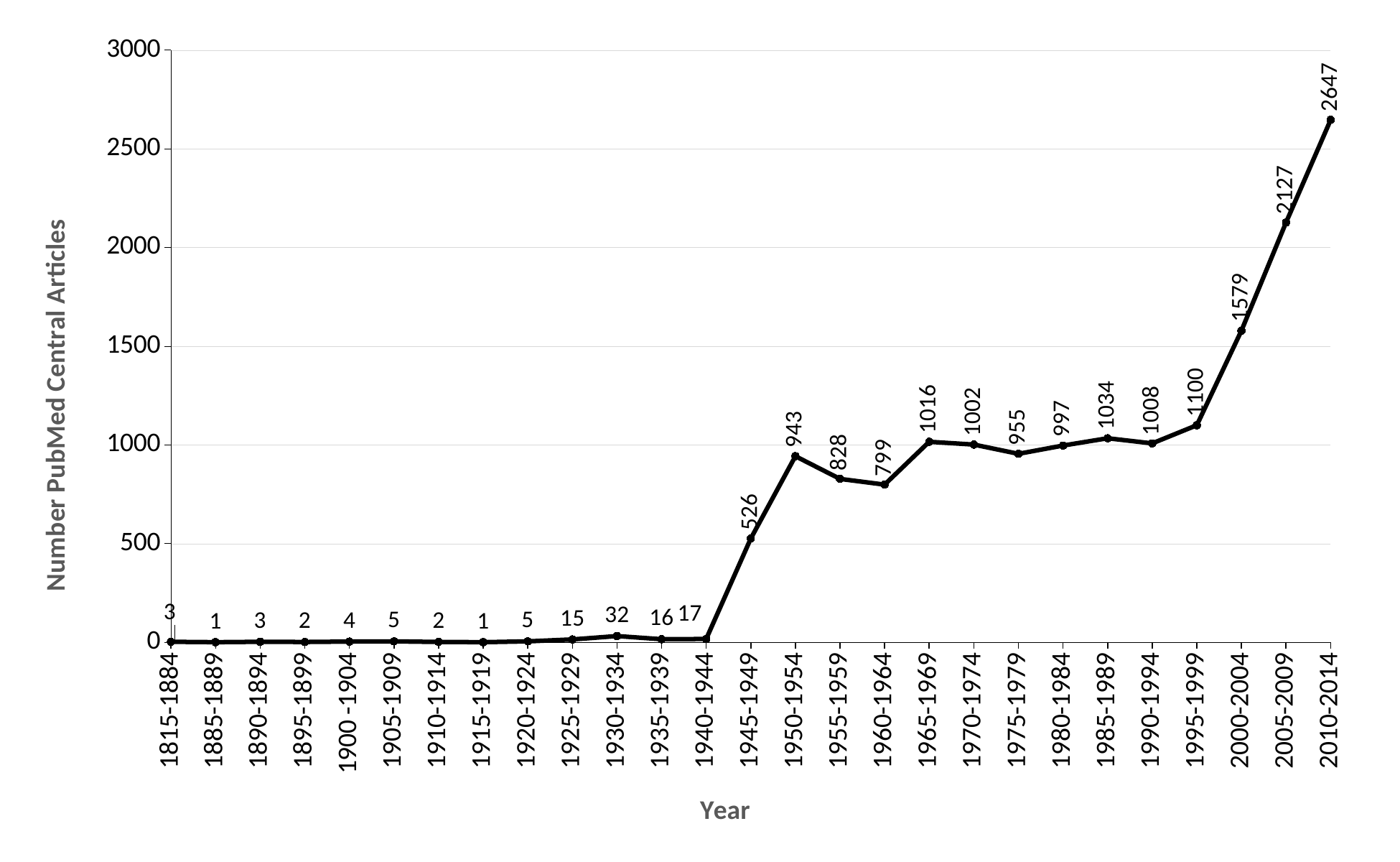

### Chart
| Category | |
|---|---|
| 1815-1884 | 3.0 |
| 1885-1889 | 1.0 |
| 1890-1894 | 3.0 |
| 1895-1899 | 2.0 |
| 1900 -1904 | 4.0 |
| 1905-1909 | 5.0 |
| 1910-1914 | 2.0 |
| 1915-1919 | 1.0 |
| 1920-1924 | 5.0 |
| 1925-1929 | 15.0 |
| 1930-1934 | 32.0 |
| 1935-1939 | 16.0 |
| 1940-1944 | 17.0 |
| 1945-1949 | 526.0 |
| 1950-1954 | 943.0 |
| 1955-1959 | 828.0 |
| 1960-1964 | 799.0 |
| 1965-1969 | 1016.0 |
| 1970-1974 | 1002.0 |
| 1975-1979 | 955.0 |
| 1980-1984 | 997.0 |
| 1985-1989 | 1034.0 |
| 1990-1994 | 1008.0 |
| 1995-1999 | 1100.0 |
| 2000-2004 | 1579.0 |
| 2005-2009 | 2127.0 |
| 2010-2014 | 2647.0 |

Supplement: Supplementary Data [file supp_bav110_suppl_data.zip › SupplemetaryFile_3.pptx]
